# A comparison of meningococcal carriage by pregnancy status

**DOI:** 10.1186/1477-5751-9-6

**Published:** 2010-08-11

**Authors:** Eric J Knudtson, Mike L Lytle, Beverly A Vavricka, Valerie S Skaggs, Jennifer D Peck, Andrew E Elimian

**Affiliations:** 1 Department of Obstetrics and Gynecology, Division of Maternal Fetal Medicine, The University of Oklahoma Health Sciences Center, PO Box 26901, WP 2470, Oklahoma City, OK, 73160 USA; 2 Oklahoma State Department of Health, 1000 NE 10th, Oklahoma City, OK 73117 USA; 3 The University of Oklahoma Health Sciences Center College of Public Health, 801 N.E. 13th St. Oklahoma City, OK 73104 USA

## Abstract

Neisseria meningitidis is the second leading cause of invasive meningitis. A prerequisite for infection is colonization of the nasopharynx, and asymptomatic carrier rates are widely reported in the range of 10-15%. Recent reports have indicated an increased likelihood that a pediatric admission for Neisseria meningitidis will have a mother who is pregnant in the home. We hypothesized that this association may relate to immunologic changes in pregnancy leading to higher carrier rates.

We compared the carrier status by performing nasopharyngeal swabs for Neisseria meningitidis in 100 pregnant and 99 non-pregnant women.

Average age of the participants was 28.9 +/- 6.7 years. The average gestational age at specimen collection was 27.5 +/- 9.4 weeks. Non pregnant women were significantly more likely to use tobacco (38% vs 24%, p < 0.0001). In the entire 199 patients, only one pregnant patient tested positive for Neisseria meningitidis (0.5%; 95% CI: 0.01%-2.8%).

The meningococcal carrier rate in our population is well below what is widely reported in the literature. Assuming a 1% carrier rate in the pregnant group and a 0.5% carrier rate in the non pregnant group, 4,763 patients would be required to detect a difference of this magnitude, given 80% power and an alpha of 0.05.

## Background

Neisseria meningitidis, simply known as meningococcus, is a gram negative bacterium and a leading cause of bacterial meningitis. Nasopharyngeal carriage is a prerequisite for invasive disease, and approximately 10-15% of healthy individuals are reported to carry the organism at any one time. Rates of carriage and transmission are known to increase in closed, or semi-closed living conditions such as military barracks, jails, and college dormitories[1,2] Additional factors shown to affect the carriage rate are: age, gender, social class, exposure to cigarette smoke, and vaccination[3,4].

Recent information demonstrates that pediatric disease may correlate with a pregnant mother. For example, van Gils et al. evaluated 176 hospitalized children, half of whom were admitted for invasive meningococcus. Amongst the cases 19% of children were found to have a mother who was pregnant, compared to only 2% of controls. Multivariate analysis showed meningococcal disease was 11.7 times more likely to occur in a child whose mother was pregnant[5].

One mechanism may be the immunologic changes of pregnancy predispose a woman to being an asymptomatic carrier. To test that hypothesis, we planned a cross-sectional observational study of nasopharyngeal carriage of meningococcus to compare rates by pregnancy status.

## Methods

This was an observational study. Institutional Review Board approval was sought and obtained. Women presenting for care at the University of Oklahoma Health Sciences Center obstetrics and gynecology clinics were invited to participate. Samples were obtained from September 23, 2008 until May 21, 2009. Eligible women included pregnant and non-pregnant women aged 18-45 years. Women were excluded if they had twins or higher order multiple gestations. Non-pregnant women were excluded if they had a pregnancy in the preceding nine months. Additional exclusion criteria included known or suspected meningococcal vaccination or antibiotic use in the preceding month.

Women who agreed to participate had their demographic information recorded and a nasopharyngeal swab performed by a research nurse. Recorded demographic information included age, race, gravidity, parity, estimated gestational age at enrollment, estimation of annual household income, exposure to cigarette smoke, number and ages of members residing in household, and occupation.

The nasal swab was performed in a standardized manner. Briefly, after explaining the procedure, an Aimes culturette swab with charcoal^® ^was passed through the nares until resistance was met. The nasopharynx was sampled for 30 seconds and the swab was removed. Specimens were labeled with a study number and the legend was kept in a separate secure location.

The specimens were then immediately transported to the Oklahoma State Department of Health where they were immediately plated on Modified Thayer Martin agar. Plates were incubated at 35-37 degrees C in 5-8% CO2 at 18-24 hours and 36-48 hours. Those specimens identified as suspect for Nisseria meningitidis were tested for gram stain and oxidase testing. Specimens that were positive for gram-negative diplococci and oxidase test positive were plated on chocolate agar to incubate for 18-24 hours. After incubation, identification of the isolate was performed using the Biomerieux Vitek API NH system. The generated code number was entered into the API software, with a result of Neisseria meningiditis with a greater than 85% probabilities was considered definitive identification. Each isolate identified as Neisseria meningitidis was plated on Heart infusion agar with 5% sheep blood and incubated for 18-24 hours. After incubation, specimens were then serotyped.

Our *null hypothesis *was that pregnant women were not more likely to be carriers of Neisseria meningitidis. The difference in proportion of carriers by pregnancy status was evaluated using a Fisher's exact chi-square test. Pearson or Fisher's Exact chi-square tests were performed to determine if the distribution of demographic characteristics differed by pregnancy status. Differences in mean age were examined using a Student's t-test. In a cross sectional study designed to evaluate college dormitory students in the United Kingdom, students beginning the fall term in October had a baseline carriage rate of 14%. One month later the carriage rate was 31%, (a 120% increase) [6].

Assuming a similar baseline carrier rate, a sample size of 200 (100 pregnant women and 100 non-pregnant women) provided for 80% power to detect a 16% to 18% absolute difference (a 120% relative increase) in carriage rates between pregnant and non-pregnant women using an alpha of 0.05.

## Results

Two-hundred and sixty one patients were evaluated and fifty five either declined to participate or were ineligible due to age or recent antibiotic use. Nasopharyngeal swabs were performed on 206 women (103 pregnant and 103 non-pregnant). The six additional cultures beyond the sample size calculation were performed after a review showed that 4 patients were swabbed despite meningococcal vaccination, and 3 samples never had final results reported by the Oklahoma State Department of Health. The final cohort consisted of 199 patients, (100 pregnant and 99 non-pregnant). Table 1 shows the demographic characteristics and descriptive statistics for the study population. The average age of all participants was 28. 9 standard deviation (sd 6.7 years). Gestational age ranged from 7 to 40 weeks, with a mean of 27.5 weeks (sd 9.4). There was a significant difference between the pregnant and non-pregnant women in terms of race and smoking status. In the entire group of 199 patients, only one carrier was identified (0.5%; 95% CI: 0.01%-2.8%). She was a 23 year old African American woman. She was pregnant and in her 39^th ^week of pregnancy. She smoked, was unemployed and was found to have meningococcal serotype group X.

**Table 1:** Characteristics of the study group

	Pregnant (n = 100) N or Mean (sd)	Nonpregnant (n = 99) N or Mean (sd)	p
Age (mean, sd)	27.60 (5.90)	30.17 (7.22)	NS^a^
Parity			
0	37	41	NS^b^
1	27	22	
2	22	18	
3	7	9	
4	3	3	
5	3	3	
6	0	2	
7	1	0	
9	0	1	
Race			
Caucasian	39	60	0.0002^c^
African American	12	20	
Hispanic	40	15	
Native American	5	2	
Asian	2	2	
Other	2	0	
Estimated Gestational Age	27.49 (9.36)	N/A	N/A
Tobacco Use	14	38	<0.0001^c^
Number in Household			
1	2	16	0.0106^c^
2	23	25	
3	26	20	
4	30	23	
>5	19	15	
Median Income			
<20,000	23	17	0.5951^c^
20,000-39,999	20	19	
40,000-59,999	7	13	
>60,000	10	11	
Unknown	39	40	

^a ^Student's t-test

^b ^Fisher's exact chi square test

^c ^Pearson's Chi-square test

## Discussion

Disease from Neisseria meningitidis remains a significant public health problem. Nasopharyngeal carriage is a prerequisite for invasive infection and the proportion of asymptomatic carriers varies based on a number of factors, with higher rates associated with increased disease incidence [2,6,7]. Recently, van Gils published work describing pediatric hospital admissions for invasive Neisseria meningitidis. Patients with meningitis were nearly 12 times as likely to have a mother who was pregnant in the household as compared to controls[5].

If true, how might pregnancy confer an increased risk to another member in the household? One plausible explanation is that pregnancy confers a transient increase in the maternal carriage of Neisseria meningitidis. Susceptible household members such as young children would then be more prone to transmission and active disease. To test this hypothesis we designed a simple observational study to compare the proportion of carriers in our pregnant and non-pregnant populations. Our sample size was based on repeated estimates of the general population carriage rate being approximately 10-15%[1].

To our surprise, the entire cohort of 199 patients showed only one positive result. There are several possible explanations. The first is that there were specimen collection and/or laboratory errors that led to a systematic error in the classification of carrier status. Our review shows this to be an unlikely explanation for these results. At the halfway point of patient recruitment, we reviewed our specimen collection, transport, and processing procedures and found them to be consistent with published reports[1,4,8]. Also, our specimens were analyzed at the Oklahoma State Department of Health, which has an experienced microbiology lab. A review of the laboratory techniques also showed no lapse in quality control. Laboratory procedures were consistent with published standards [1,4,8] (Michael Lytle, OSDH, personal communication).

The more likely explanation appears to be that meningococcal carriage has decreased significantly over time. This can be reasonably inferred by looking at the disease incidence in the state of Oklahoma. Over a twelve year time span the meningococcal disease incidence rate decreased from approximately 1.5 to 0.5/100,000[9].

Additionally, a requested review of results at the microbiology lab at the University of Oklahoma showed that in the 15 months prior to completing this study, there was not one report of an incidental finding of Neisseria meningitidis from any nasopharyngeal cultures taken for routine clinical care [personal communication, Mary Magnus, director, OUHSC microbiology lab].

The underlying mechanism for the lower than expected carriage rate cannot be explained by this study. Carriage, transmission and disease burden vary naturally over time. Additionally, use of the MC4 vaccine has expanded. In particular, the change in the recommendations of the Advisory Committee on Immunization Practices, which expanded vaccination to all adolescents, may influence the carriage rate in the general population. While vaccination was an exclusion criterion for participation in this study, a herd immunity effect is possible.

Finally, our decision to use nasopharyngeal cultures may also have had an impact on our carrier rate. In some reports, the nasopharyngeal culture resulted in a slightly lower sensitivity when compared to the oropharyngeal technique[10].

## Conclusions

In summary, there was no significant difference in carriage of Neisseria meningococcus in relation to pregnancy status. Only one carrier was identified in the entire study population of 199 patients. Thus, the 0.5% (95% CI: 0.01%-2.8%) carriage rate observed among women at the University of Oklahoma obstetrics and gynecology clinics is well below estimates reported for other populations.

## Competing interests

The authors declare that they have no competing interests.

## Authors' contributions

EJK conceived the study, designed the experiment, helped with data acquisition and analysis and wrote the manuscript. MLL performed the microbiologic cultures. BAV was primarily responsible for patient recruitment and culture sampling. VSS assisted with study design, and performed data analysis. JDP assisted with study design, data analysis and manuscript preparation. AEE assisted with manuscript preparation. All authors wrote and approved the final manuscript.
